# Machine learning-driven multi-omics integration uncovers a senescence associated molecular axis in HCC

**DOI:** 10.3389/fimmu.2026.1762222

**Published:** 2026-05-08

**Authors:** Talaiti Tuergan, Aimitaji Abulaiti, Yierfan Yilihaer, Bingwei Liu, Yingmei Shao, Tiemin Jiang, Tuerganaili Aji

**Affiliations:** 1 Hepatobiliary and Echinococcosis Surgery Department, Digestive and Vascular Surgery Center, First Affiliated Hospital of Xinjiang Medical University, Urumqi, China; 2 State Key Laboratory of Pathogenesis, Prevention and Management of High Incidence Diseases in Central Asia, Xinjiang Medical University, Urumqi, China

**Keywords:** cellular senescence, hepatocellular carcinoma, MCM7, multi-omics, random survival forest

## Abstract

**Background:**

Hepatocellular carcinoma (HCC) exhibits profound molecular heterogeneity and aberrant cellular senescence. This study systematically dissects the senescence-associated molecular landscape to identify key regulators driving HCC progression and immune evasion.

**Methods:**

Integrating multi-cohort transcriptomic datasets, we developed a robust prognostic signature using 101 machine-learning models, identifying prognostic signature. We employed preliminary proteomic, exploratory metabolomic, and single-cell RNA sequencing (scRNA-seq) analyses to explore multi-omics alterations. The functional senescence status and MCM7 were validated in a clinical HCC cohort by RT–qPCR, Western blotting, immunohistochemistry, and multiplex immunofluorescence (mIF). Causality was established using *in vitro* functional assays in HepG2 cells.

**Results:**

A 12-gene random survival forest (RSF) signature accurately predicted patient survival across independent cohorts. MCM7 emerged as a central senescence-associated driver. ScRNA-seq and mIF confirmed MCM7 characterizes a highly proliferative, clonally expanding subset of CD8^+^ T cells within the tumor microenvironment. *In vitro*, MCM7 knockdown significantly inhibited HepG2 cell proliferation and upregulated senescence enforcers p16 and p21, whereas overexpression facilitated evasion. Additionally, TIDE analysis revealed that high-risk patients exhibited elevated immune evasion potential, predicting poor immunotherapy response.

**Conclusion:**

This integrative multi-omics framework uncovers an MCM7 MCM7-driven senescence-associated axis promising HCC progression and immune dysfunction, offering a robust tool for prognostic stratification and novel therapeutic insights.

## Introduction

Hepatocellular carcinoma (HCC) remains a major global health burden, with more than 900,000 new cases and over 800,000 deaths reported in 2020 (1). Its incidence is particularly high in regions with chronic hepatitis B and C infection, alcohol-related liver disease, and metabolic dysfunction–associated steatotic liver disease (2). Despite improvements in surveillance and therapy, the 5-year survival rate of HCC remains below 20% (3), highlighting the need for better molecular markers to guide prognosis and treatment.

HCC exhibits profound molecular heterogeneity driven by diverse etiologies, genetic alterations and microenvironmental pressures (4). Multi-omics studies have defined several molecular subclasses of HCC, yet most existing markers primarily focus on proliferation, immune activation or metabolic traits (5). Cellular senescence is traditionally defined as a durable state of growth arrest mediated by p53/p21 and p16/RB pathways, functioning as a potent tumor-suppressive barrier in early carcinogenesis (6, 7). However, growing evidence indicates that many aggressive tumors, including HCC, frequently disable or bypass these senescence-enforcing pathways to maintain proliferative capacity (8, 9). In this context, senescence in HCC is not characterized by the accumulation of stable senescent cells, but rather by the selective suppression of senescence checkpoints, enabling tumor cells to evade stress-induced arrest while exploiting certain senescence-related transcriptional programs to support metabolic and microenvironmental remodeling.

In this study, we integrated multi-cohort transcriptomic analyses, machine-learning modeling, proteomic and metabolomic profiling, single-cell RNA sequencing and clinical validation to construct a comprehensive senescence-related landscape of HCC. Our goal was to identify key senescence-associated genes, define their prognostic value, and elucidate how they link cell-cycle regulation, metabolism and the tumor microenvironment.

## Materials and methods

### Data acquisition and preprocessing

The transcriptomic datasets GSE14520, GSE76427, and the single-cell dataset GSE242889 were obtained from the Gene Expression Omnibus (GEO, https://www.ncbi.nlm.nih.gov/geo/). The RNA-seq dataset for TCGA-LIHC and its related clinical data were obtained from The Cancer Genome Atlas (TCGA, https://portal.gdc.cancer.gov/). The raw data were standardized using either the ‘limma’ or ‘DESeq2’ R packages, based on the platform type. Genes associated with cellular senescence (CSRGs) were sourced from the CellAge database (Human Ageing Genomic Resources, Senescence Genes Database, build 2; https://genomics.senescence.info/cells/). We unified all gene identifiers to standard official gene symbols and removed any duplicate entries.

GSE14520 comprises 225 HCC tumor samples and 220 normal liver samples. GSE76427 consists of 115 HCC tumor samples and 52 normal liver samples. GSE242889 includes 5 HCC samples and 5 adjacent non-tumor samples. The TCGA dataset comprises 373 HCC tumor samples and 50 normal liver samples.

### Bioinformatics analyses

Using ‘limma’ for GSE14520 and GSE76427, and ‘DESeq2’ for TCGA, differentially expressed genes (DEGs) between tumor and nearby normal liver tissues (FC > 1, adjust P value < 0.05) were identified. The Kyoto Encyclopedia of Genes and Genomes (KEGG) enrichment analyses were conducted with ‘clusterProfiler’, considering P < 0.05 as significant. ‘ggplot2’ was used to create bubble and bar plots.

Then a total of 101 machine-learning survival models (10) were constructed using combinations of random survival forest (RSF), elastic network (Enet), Lasso, Ridge, stepwise Cox, CoxBoost, partial least squares regression for Cox (plsRcox), supervised principal components (SuperPC), generalized boosted regression modeling (GBM), and survival support vector machine (survival-SVM). To guarantee reproducibility, a random seed (seed = 123) was set for all computational steps. To ensure robust model construction and prevent data leakage, the datasets were strictly split into independent phases. The GSE14520 cohort was designated solely as the internal training set for feature selection and model training, while the TCGA and GSE76427 cohorts served as strictly independent external testing sets. For hyperparameter tuning, we employed a 10-fold cross-validation nested strictly within the training set to prevent overfitting. For the optimal model, a grid search via 10-fold cross-validation within the training set was utilized to determine the optimal variables randomly sampled as candidates at each split (mtry) and minimum node size (nodesize), with the final core parameters set to ntree = 1000 and nodesize=5. No testing set data were exposed during the feature selection or hyperparameter tuning phases. The optimal model-gene signature was used to calculate individual risk scores.

Ultimately, the optimal model-gene signature was used to calculate individual risk score. To stratify patients into high- and low-risk groups, the optimal cutpoint for the risk score was calculated utilizing the maximally selected rank statistics via the maxstat R package (version 0.7-25). To ensure statistical stability, the minimum and maximum sample proportions in each group were restricted to >25% and <75%, respectively. Crucially, this optimal cutoff value (Cutoff = 22.88) was determined exclusively within the internal training cohort (GSE14520), and applied to stratify patients in both independent external testing cohorts (GSE76427 and TCGA). To evaluate the potential clinical translational value of the prognostic signature, the Tumor Immune Dysfunction and Exclusion (TIDE) algorithm (http://tide.dfci.harvard.edu/) was utilized. Higher TIDE scores typically indicate a higher likelihood of immune escape and a lower probability of responding to immune checkpoint blockade (ICB) therapy.

Predictive performance was evaluated using the concordance index (C-index) alongside 1-, 3-, and 5-year time-dependent AUCs using “timeROC” packages. Multivariable Cox regression analysis, integrating the derived risk score with core clinical covariates (age, gender, stage), was conducted to confirm the independent prognostic value of the signature in TCGA. Furthermore, univariate Cox regression analysis was used to screen for genes significantly associated with overall survival (OS, P < 0.05). Nomograms and calibration curves were generated using “rms”.

### Clinical tissue collection

Fresh human liver cancer tissues (n = 30) and matched adjacent non-tumor tissues (n = 30) were collected from patients undergoing surgical resection at First Affiliated Hospital of Xinjiang Medical University. All cases were pathologically confirmed as HCC according to WHO classification criteria. Non-tumor tissues were sampled at least 2–3 cm away from the tumor margin to avoid microscopic infiltration. Immediately after surgical removal, each specimen was divided into three portions: (1) snap-frozen in liquid nitrogen for RNA and protein extraction (n = 10), (2) fixed in 4% paraformaldehyde for histological analyses (n = 10), and (3) stored at –80 °C for proteomic and metabolomic profiling (n = 10). All clinical data were anonymized before analysis.

Inclusion criteria: Adult patients undergoing curative-intent hepatectomy. Histologically confirmed primary hepatocellular carcinoma. Availability of paired tumor and adjacent non-tumor liver tissues. No anti-cancer treatments prior to surgery, including chemotherapy, radiotherapy, targeted therapy, immunotherapy, or TACE. Exclusion criteria: Diagnosis of mixed-type liver cancer (HCC–ICC) or metastatic liver tumors. History of preoperative anti-tumor treatments (TACE, RFA, sorafenib, immunotherapy, etc.). Severe infection, autoimmune disease, or chronic inflammatory disorders. Coexistence of other malignant tumors. Poor-quality samples (e.g., severe necrosis affecting RNA/protein integrity). The study protocol was approved by the Institutional Ethics Committee of First Affiliated Hospital of Xinjiang Medical University (Approval No: K202510-27). All procedures followed the Declaration of Helsinki (2013 revision). All patients signed informed consent prior to enrollment.

### Proteomic profiling of clinical tissues

Proteomic analysis was performed on paired human HCC and adjacent non-tumor normal tissues (n = 9 pairs, poor-quality samples were excluded). Protein was extracted from frozen tissue using RIPA lysis buffer supplemented with protease and phosphatase inhibitors, and protein concentration was quantified using the BCA Protein Assay Kit (Beyotime). Proteins were then digested by trypsin and labeled with TMT 10-plex reagents. Pooled TMT-labeled peptides were fractionated using high-pH reversed-phase liquid chromatography (RP-HPLC) on an Agilent 1260 Infinity system. Liquid chromatography–tandem mass spectrometry (LC–MS/MS) was performed using a Thermo Scientific Orbitrap Fusion Lumos mass spectrometer coupled to an EASY-nLC 1200 nano-UPLC system. Raw files were processed using Proteome Discoverer (v2.4) with the Sequest HT algorithm against the Uniprot Homo sapiens database. Differentially expressed proteins (DEPs) between HCC and adjacent tissues were defined as FC > 1.2 or < 0.83 with *P* < 0.05. KEGG analysis was performed using “clusterProfiler”.

### Untargeted metabolomics

Untargeted metabolomic profiling was performed on paired HCC and adjacent non-tumor normal tissues (n = 10 pairs) using an ultra-high-performance liquid chromatography–Orbitrap mass spectrometry (UHPLC–Orbitrap MS) platform. For each sample, metabolites were extracted using methanol/acetonitrile/water (2:2:1), followed by centrifugation and vacuum drying. Raw MS data were normalized to the total ion current (TIC) and median-scaled to correct for batch variations. Quality control (QC) samples were used to evaluate system stability and data reliability. Positive and negative ion modes were analyzed. PLS-DA was performed using SIMCA software. To strictly rule out overfitting, the PLS-DA models were validated using a 200-iteration permutation test. Differential metabolites were selected based on VIP > 1 and *P* < 0.05. The confidence levels of key metabolite identifications were assigned according to the Metabolomics Standards Initiative (MSI) guidelines (Level 1/2).

### Single-cell RNA-seq analysis

Single-cell RNA sequencing (scRNA-seq) data from the GEO dataset GSE242889 were performed using Seurat (v4.3.0) in R. Low-quality cells were removed according to the following criteria: <200 or >6,000 detected genes, >10% mitochondrial gene content. Normalization was performed using SCTransform, which applies regularized negative binomial regression to correct for sequencing depth and technical noise. The top 2,000 highly variable genes (HVGs) were selected for downstream analysis. Graph-based clustering was performed using Seurat, cluster markers were identified using FindAllMarkers function. Cell types were annotated using cluster markers and the “SingleR” package. Expression visualization of genes was performed using “DotPlot” package. Cell cycle phase assignment was performed using the CellCycleScoring function in Seurat. Each cell was assigned a continuous S-score and G2M-score based on the expression of canonical cell cycle markers, and subsequently categorized into G1, S, or G2M phases.

### RT–qPCR and western blotting

Total RNA was extracted using TRIzol reagent (Invitrogen). cDNA synthesis was performed using a reverse transcription kit (Takara). qPCR was conducted using SYBR Green Master Mix (Vazyme) on a QuantStudio 6 Flex system. β-actin served as the internal control. Relative expression was calculated by the 2^-ΔΔCt^ method.

Total proteins were extracted using RIPA buffer with protease inhibitors. Equal amounts of proteins were separated by SDS-PAGE and transferred to PVDF membranes. Membranes were blocked with 5% non-fat milk and incubated overnight at 4 °C with primary antibodies against CDKN1A (p21), CDKN2A (p16), MCM7, and β-actin (Proteintech, China). After incubation with HRP-conjugated secondary antibodies, signals were detected using ECL reagents.

### Immunohistochemistry

Paraffin-embedded tissues were sectioned (4 μm), deparaffinized, and subjected to antigen retrieval using citrate buffer (pH 6.0). Sections were incubated with primary antibodies against CDKN1A, CDKN2A, MCM7, and lactyl lysine followed by HRP-labeled secondary antibodies. DAB chromogen was used for visualization. Senescence-Associated β-galactosidase (SA-β-gal) were detected using frozen section within stain kit (YEASEN, China). The percentage of positive staining area were calculated using ImageJ.

### Multiplex Immunofluorescence

To validate the co-expression of MCM7 in CD8+ T cells at the protein level, multiplex immunofluorescence (mIF) staining was performed on paraffin-embedded HCC tissue sections. Following deparaffinization, rehydration, and antigen retrieval, the sections were blocked and incubated overnight at 4 °C with a mixture of primary antibodies against MCM7 and CD8. After extensive washing, the sections were incubated with corresponding fluorescently-conjugated secondary antibodies. Nuclei were counterstained with DAPI. Fluorescent images were captured using a fluorescence microscope to evaluate the spatial co-localization of MCM7 and CD8 in tumor-infiltrating lymphocytes.

### Cell culture and transfection

The human HCC cell line HepG2 was obtained from the Procell (China) and cultured in DMEM supplemented with 10% fetal bovine serum (FBS) and 1% penicillin/streptomycin at 37 °C in a humidified 5% CO_2_ atmosphere. To knock down MCM7 expression, HepG2 cells were transfected with MCM7-specific small interfering RNA (si-MCM7) or a negative control (NC) using Lipofectamine 3000 (Invitrogen) according to the manufacturer’s instructions. For overexpression, an MCM7 overexpression plasmid (oe-MCM7). Cells were harvested 48 hours post-transfection for downstream functional and molecular assays.

### Cell proliferation assay

Cell proliferation was evaluated using the Cell Counting Kit-8 (CCK-8, Beyotime, China). Briefly, transfected HepG2 cells were seeded into 96-well plates at a density of 2000 cells per well. At specified time points (24, 48, 72, and 96 hours), 10 μL of CCK-8 reagent was added to each well and incubated for 2 hours at 37 °C. The optical density (OD) at 450 nm was measured using a microplate reader.

### Statistical analysis

Statistical analyses were performed using R (v4.3.0) and GraphPad Prism 10.0.3 Continuous variables were compared using Student’s *t*-test or Wilcoxon test. Correlation analyses used Spearman coefficients. Survival curves were compared using the log-rank test. *P* < 0.05 was considered statistically significant.

## Results

### Multi-cohort transcriptomic profiling identifies cellular senescence–related dysregulated genes in HCC

**Figure 1:**
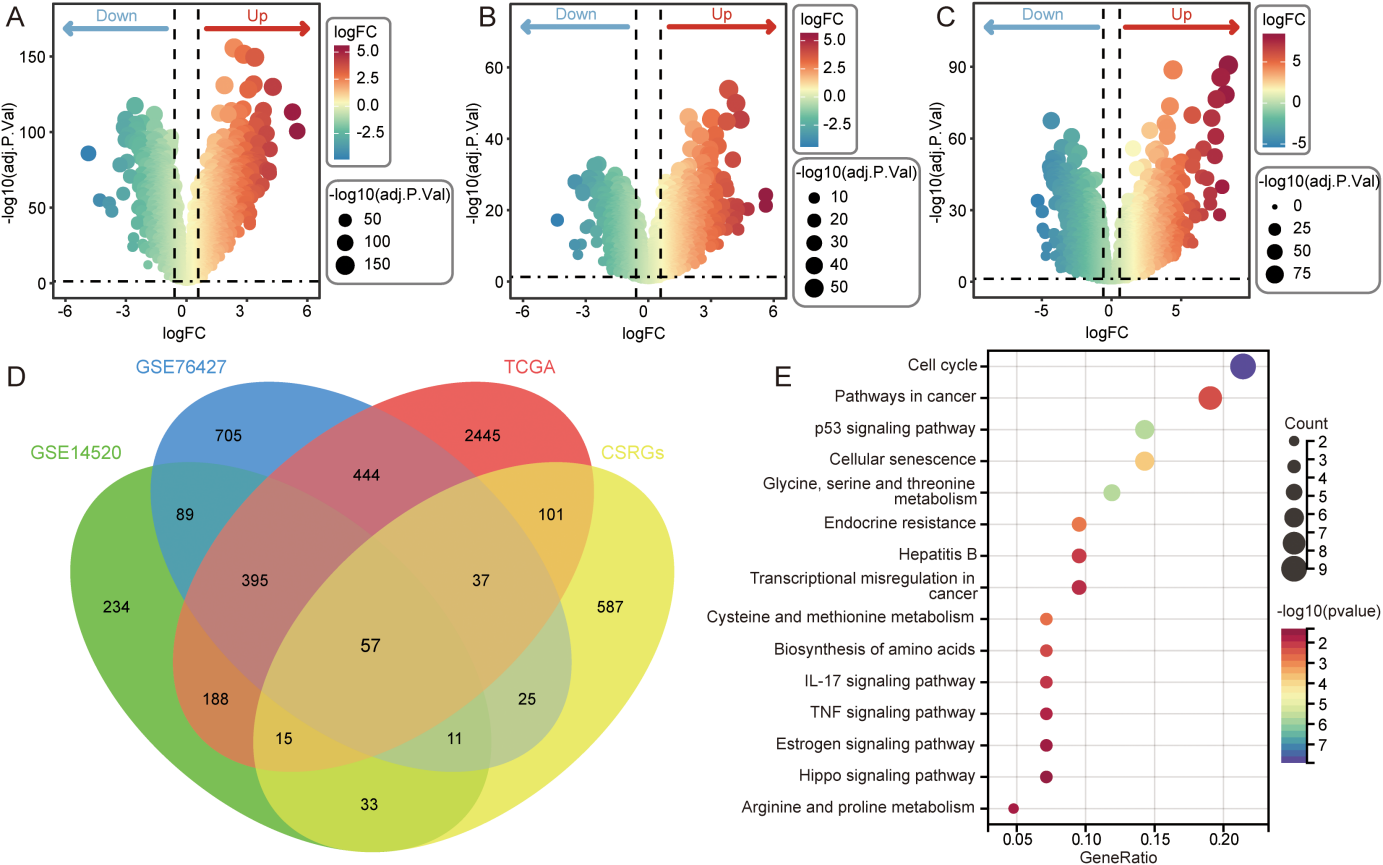
Identification of cellular senescence–related DEGs in HCC. **(A–C)** volcano plots showing differentially expressed genes (DEGs) between HCC and adjacent normal tissues in GSE14520 (1022 DEGs), GSE76427 (1763 DEGs), and TCGA-LIHC (3682 DEGs). **(D)** Venn diagram showing the intersection of DEGs from three cohorts with curated cellular senescence-related genes (CSRGs). **(E)** KEGG enrichment analysis of the 57 overlapping genes.

To systematically characterize senescence-associated transcriptional alterations in HCC, differential expression analysis was performed across three independent cohorts (GSE14520, GSE76427, and TCGA). We identified 1022 DEGs in GSE14520 (**Figure 1A**), 1763 DEGs in GSE76427 (**Figure 1B**), and 3682 DEGs in TCGA (**Figure 1C**). Intersection of these DEGs with a curated panel of CSRGs yielded 57 overlapping genes (**Figure 1D**), indicating a strong senescence-associated transcriptional reprogramming in HCC. KEGG enrichment analysis revealed significant enrichment of these overlapping genes in biological pathways essential for tumorigenesis and senescence-related, including cell cycle, p53 signaling pathway, pathways in cancer, cellular senescence, and glycine, serine and threonine metabolism (**Figure 1E**). These findings suggest that dysregulated senescence programs may serve as important drivers of cell-cycle deregulation and metabolic remodeling in HCC.

### Machine-learning integration identifies a robust 12-gene RSF prognostic signature

**Figure 2:**
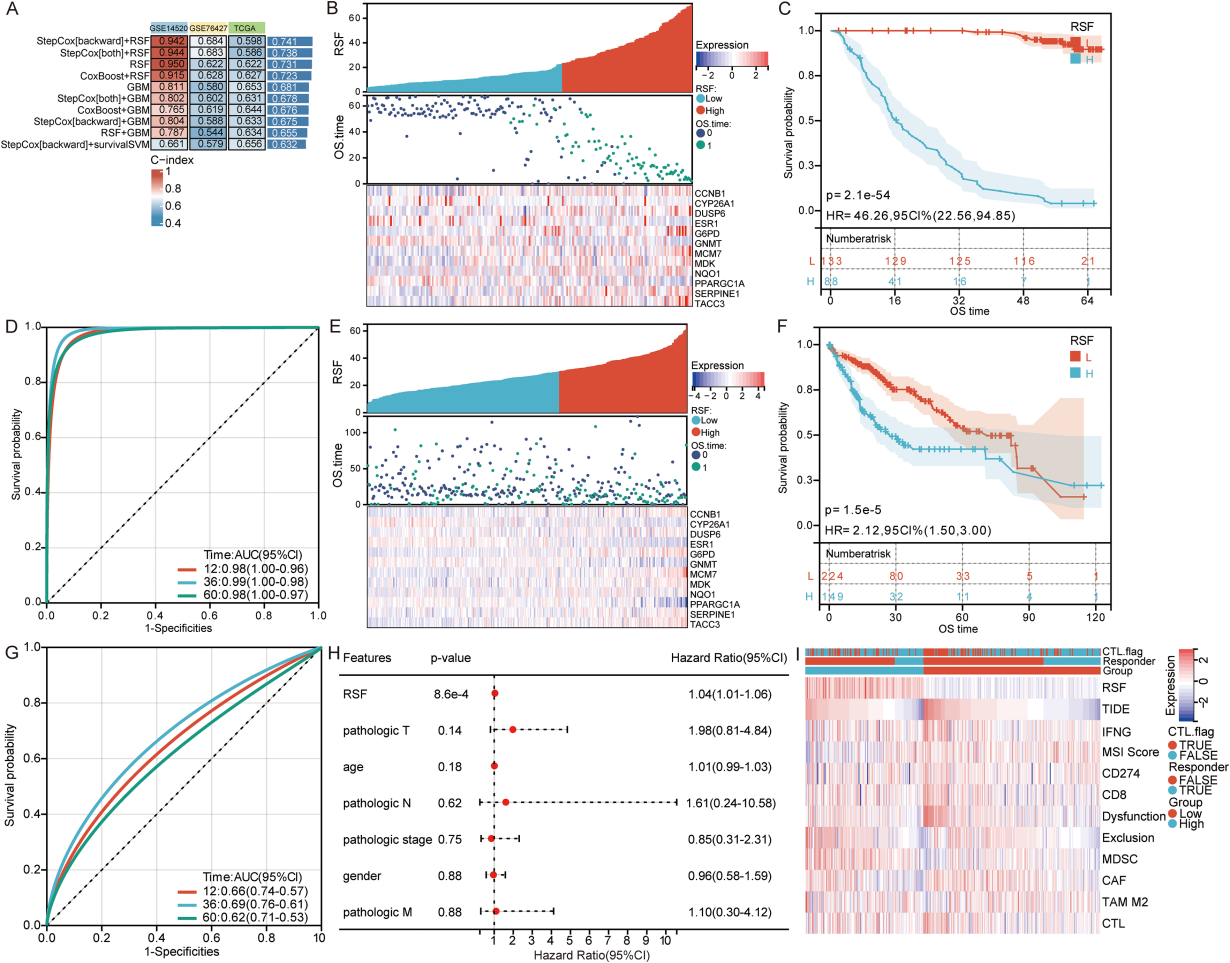
Machine-learning identifies prognostic signature. **(A)** Performance matrix of the top machine-learning models across the internal training (GSE14520) and external testing cohorts (GSE76427, TCGA-LIHC), evaluated by the C-index. **(B)** Risk score distribution, survival status, and expression heatmap of the 12-gene signature in the GSE14520 training cohort. **(C)** Kaplan–Meier survival curve of high- and low-risk patients in the GSE14520 cohort. **(D)** Time-dependent ROC curves predicting 1-, 3-, and 5-year (12, 36, and 60 months) overall survival in the GSE14520 cohort. **(E)** Risk score distribution, survival status, and expression heatmap in the TCGA testing cohort. **(F)** Kaplan–Meier survival curve in the TCGA testing cohort. **(G)** Time-dependent ROC curves at 1-, 3-, and 5-years in the TCGA testing cohort. **(H)** Forest plot of the multivariable Cox regression analysis. **(I)** Violin plot comparing the TIDE (tumor immune dysfunction and exclusion) scores between the high-risk and low-risk groups.

A total of 101 machine-learning survival models were established using combinations of 10 algorithms for 57 overlapping genes within the training cohort (GSE14520) and two independent external testing cohorts (GSE76427, TCGA). Among these models, StepCox(backward)+RSF, StepCox(both)+RSF, RSF alone, and CoxBoost+RSF achieved the highest stability, each yielding a mean C-index > 0.7 across all cohorts (**Figure 2A**). Given its optimal balance of predictive performance and model simplicity, the standalone RSF model was selected, deriving a 12-gene prognostic signature: CCNB1, CYP26A1, DUSP6, ESR1, G6PD, GNMT, MCM7, MDK, NQO1, PPARGC1A, SERPINE1, and TACC3 (**Supplementary Figure 1**). Risk score calculated from this 12-gene RSF signature effectively stratified patient survival. In the GSE14520 training cohort, patients in the high-risk group exhibited markedly worse overall survival (**Figures 2B, C**), and the time-dependent AUCs for predicting 1-, 3-, and 5-year (12, 36, and 60 months) survival were 0.98, 0.99, and 0.98, respectively (**Figure 2D**). This robust stratification was successfully validated in the independent TCGA testing cohort, where the high-risk group also showed significantly poorer survival (**Figures 2E, F**), with 1-, 3-, and 5-year AUCs of 0.66, 0.69, and 0.62, respectively (**Figure 2G**). Crucially, to determine whether the RSF signature could serve as an independent prognostic factor, we performed a multivariable Cox regression analysis incorporating the risk score alongside core clinical covariates, including age, gender, pathologic T, N, M, and overall stage. The analysis confirmed that the RSF-derived risk score remained a highly significant and independent risk factor for overall survival (Hazard Ratio = 1.04, 95% CI: 1.01-1.06), unaffected by traditional clinical parameters (**Figure 2H**). Using the TIDE algorithm, we compared the immune evasion potential between the distinct risk groups. As shown in **Figure 2I**, patients in the high-risk group exhibited significantly elevated TIDE scores compared to the low-risk group, predicting a poorer clinical response to standard ICB therapies.

**Figure 3:**
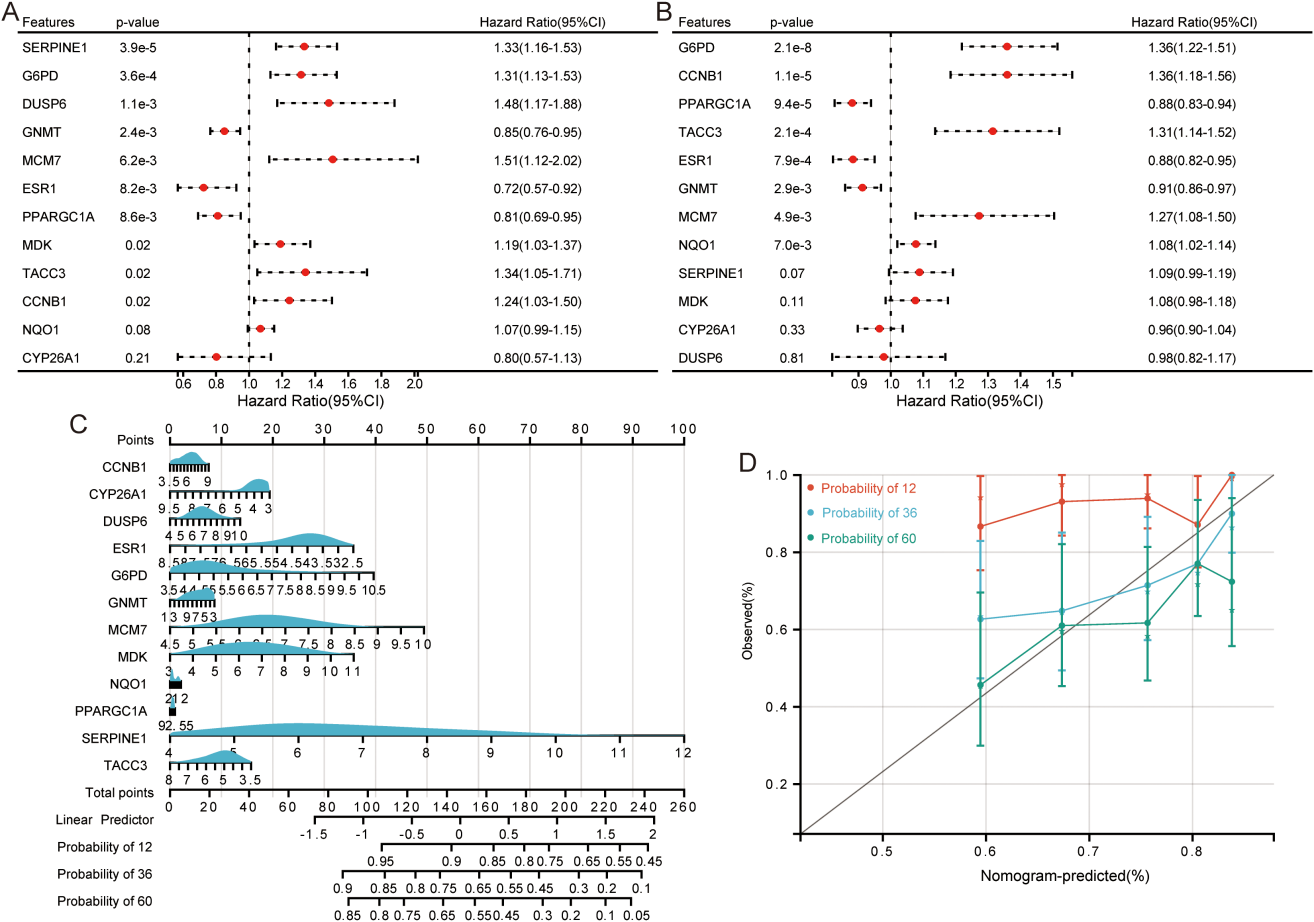
Cox regression and nomogram analysis identify MCM7 as the top contributor to HCC prognosis. **(A)** Univariate Cox regression of 12-gene signature in the GSE14520 cohort. **(B)** Univariate Cox regression of 12-gene signature in the TCGA-LIHC cohort. **(C)** Nomogram integrating the 12-gene signature for individualized survival prediction. **(D)** Calibration curves showing high concordance between predicted and observed 1-, 3-, and 5-year survival probabilities.

Univariate Cox regression further identified G6PD, CCNB1, TACC3, MCM7 as risk factors and GNMT, ESR1, PPARGC1A as protective factors in GSE14520 (**Figure 3A**), and TCGA datasets (**Figure 3B**). Kaplan–Meier survival curves supported these findings (**Supplementary Figures 2A, B**). Nomogram analysis revealed MCM7 as the top contributor to the prognostic model (**Figure 3C**), and calibration plots demonstrated excellent predictive accuracy (**Figure 3D**).

### Preliminary proteomic analysis suggests four HCC-associated senescence-related proteins

**Figure 4:**
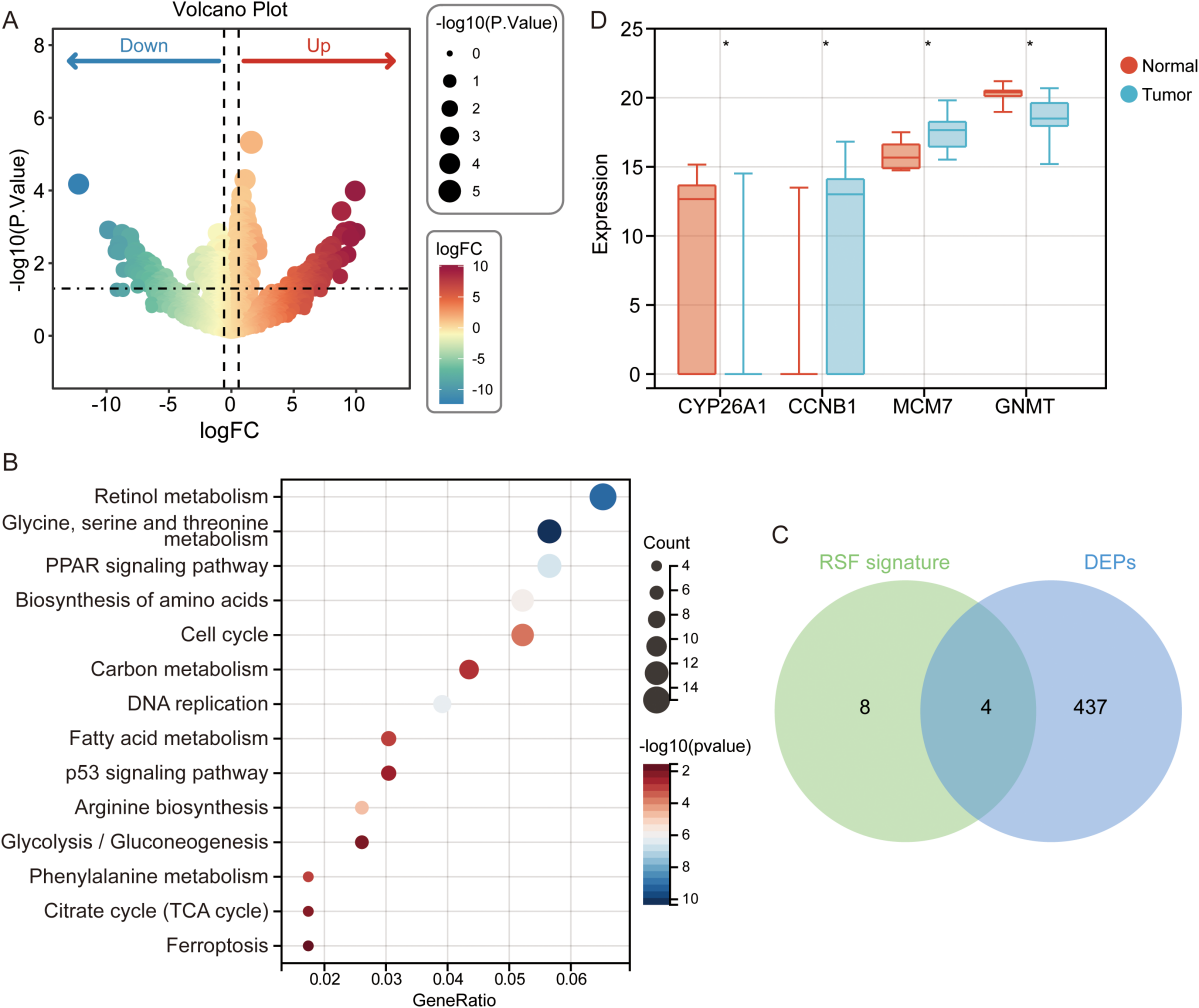
Proteomic profiling highlights four HCC-associated senescence-related proteins. **(A)** Volcano plot displaying differentially expressed proteins (DEPs) between HCC and paired normal tissues. **(B)** KEGG pathway enrichment analysis of DEPs. **(C)** Intersection of DEPs with the 12-gene signature. **(D)** Protein expression levels of CYP26A1, CCNB1, MCM7, and GNMT in HCC vs. normal tissues.

Proteomic profiling of HCC and normal tissues revealed 441 DEPs (**Figure 4A**; **Supplementary Table 1**). KEGG enrichment identified pathways regulating retinol metabolism, glycine/serine/threonine metabolism, PPAR signaling, amino acid biosynthesis, and cell cycle regulation (**Figure 4B**). Integrating proteomic DEPs with the 12-gene RSF signature identified four overlapping proteins—CYP26A1, CCNB1, MCM7, and GNMT (**Figure 4C**). Expression analysis demonstrated that CCNB1 and MCM7 were significantly up-regulated in HCC, whereas CYP26A1 and GNMT were markedly down-regulated (**Figure 4D**), consistent with transcriptomic findings.

### Exploratory metabolomic profiling reveals distinct metabolic states and gene–metabolite correlations in HCC

**Figure 5:**
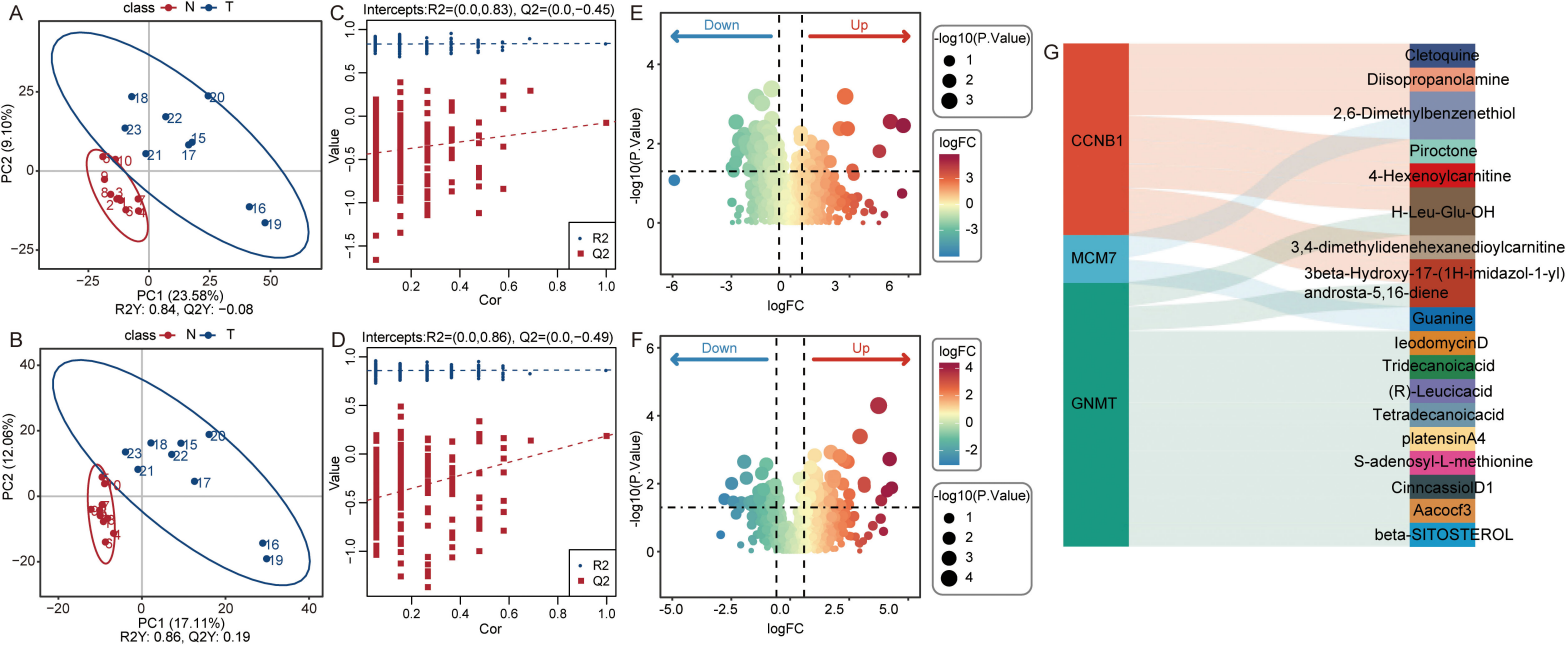
Untargeted metabolomics reveals distinct metabolic states and gene–metabolite correlations in HCC. **(A, B)** PLS-DA score plots demonstrating clear discrimination between HCC (T) and normal (N) tissues in positive **(A)** and negative **(B)** ion modes. **(C, D)** validation of the PLS-DA models via 200-iteration permutation tests in positive **(C)** and negative **(D)** ion modes. The negative Q^2^ intercepts confirm that the models are not overfitted. **(E, F)** volcano plots showing significantly differentially expressed metabolites in positive **(E)** and negative **(F)** ion modes. **(G)** Sankey diagram illustrating the significant associations between key RSF genes (CCNB1, MCM7, GNMT) and specific differential metabolites.

Untargeted metabolomics revealed a clear separation between tumor and normal samples in both positive (**Figure 5A**) and negative (**Figure 5B**) ion modes using PLS-DA. To rigorously validate the robustness of this metabolic discrimination and rule out overfitting, 200-iteration permutation tests were conducted. The results demonstrated robust model validity with negative Q^2^ intercepts in both positive (R^2^ intercept = 0.0, Q^2^ intercept = -0.45; **Figure 5B**) and negative (R^2^ intercept = 0.0, Q^2^ intercept = -0.49; **Figure 5D**) ion modes. Differential metabolite screening identified numerous significantly altered metabolites across both positive (**Figure 5E**) and negative (**Figure 5F**) ion modes. Correlation analysis (Sankey diagram) highlighted tight intrinsic links between specific metabolic shifts and key RSF genes (CCNB1, MCM7, and GNMT) (**Figure 5G**), indicating a robust coupling between MCM7 expression and global metabolic reprogramming in HCC.

### ScRNA-seq reveals cell-type–specific expression of MCM7 in tumor-associated immune cells

**Figure 6:**
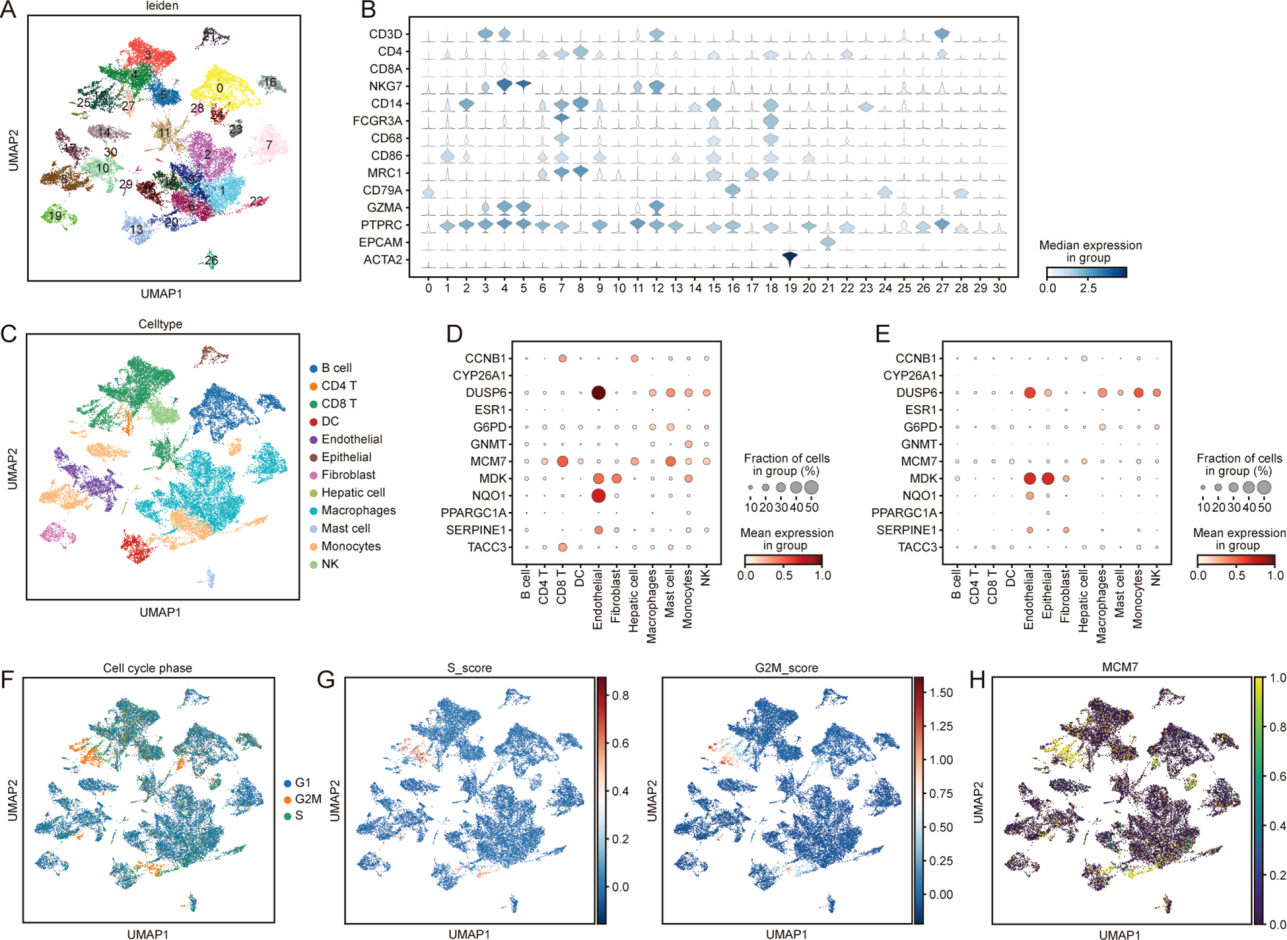
scRNA-seq analysis reveals cell-type. **(A)** UMAP visualization showing 31 clusters from the GSE242889 scRNA-seq dataset. **(B)** Expression of canonical cell-type marker expression. **(C)** Cell-type annotation of clusters, identifying B cells, CD4 T cells, CD8 T cells, NK cells, dendritic cells, macrophages, monocytes, mast cells, endothelial cells, epithelial cells, fibroblasts, and hepatocytes. DotPlot showing expression of MCM7 across cell types in tumor **(D)** and normal **(E)** Samples. **(F)** UMAP plot colored by cell cycle phase (G1, S, and G2M), demonstrating the distribution of proliferative cells across the clusters. **(G)** UMAP plots displaying the continuous S score and G2M score. **(H)** UMAP plot showing the MCM7-enriched clusters correspond to S states.

ScRNA-seq dataset GSE242889 was reanalyzed to characterize the cellular distribution of CSRGs. A total of 31 clusters were identified (**Figure 6A**) and annotated into B cells, CD4 T cells, CD8 T cells, NK cells, dendritic cells, macrophages, monocytes, mast cells, endothelial cells, epithelial cells, fibroblasts, and hepatocytes based on canonical markers (**Figures 6B, C**). Among the 12 RSF genes, MCM7 exhibited highly specific enrichment in tumor-derived CD8+ T cells and mast cells (**Figures 6D, E**), suggesting that MCM7 may influence immune exhaustion, activation, or proliferation within the HCC microenvironment. Given that MCM7 is a canonical DNA replication licensing factor, we rigorously evaluated whether this expression pattern was driven by cell-cycle dynamics. We performed cell cycle scoring to map the G1, S, and G2M phases (**Figure 6F**), alongside continuous S and G2/M scores (**Figure 6G**), across the single-cell landscape. We observed a striking spatial overlap between the MCM7-enriched immune clusters and the regions exhibiting high S scores (**Figure 6H**).

### Clinical validation confirms senescence-associated phenotypic alterations and MCM7 upregulation in HCC tissues

**Figure 7:**
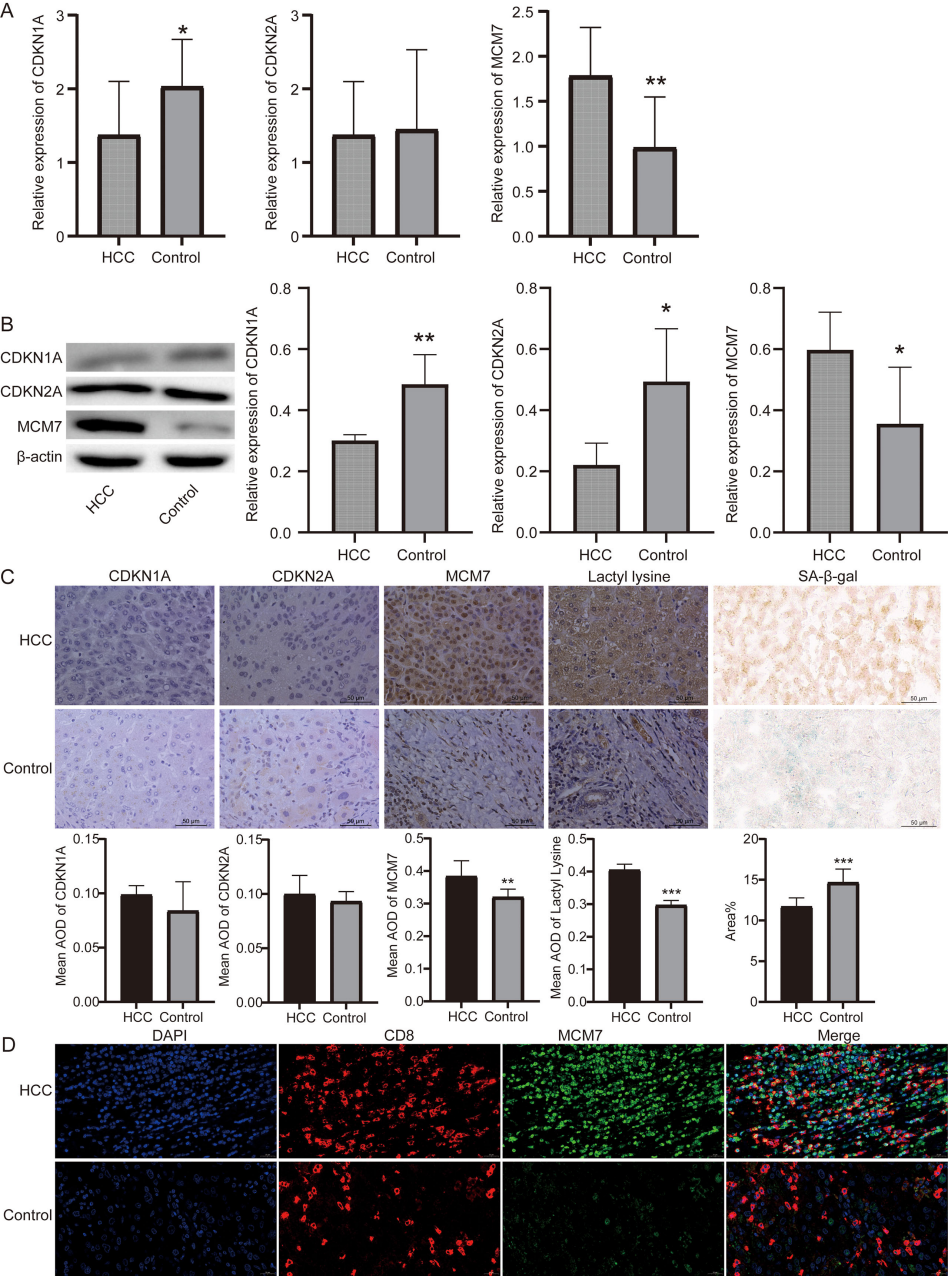
Clinical validation of MCM7, senescence markers, and metabolic rewiring in HCC tissues. **(A)** RT-qPCR analysis the mRNA levels of CDKN1A (p21), CDKN2A (p16), and MCM7 in HCC tissues and adjacent controls. **(B)** Western blot analysis and corresponding densitometry quantification of CDKN1A (p21), CDKN2A (p16), and MCM7 protein levels. **(C)** Representative images and quantitative analysis (mean AOD or area%) of immunohistochemistry (IHC) for CDKN1A, CDKN2A, MCM7, lactyl lysine, and senescence-associated β-galactosidase (SA-β-gal) staining in HCC and normal tissues. Scale bars = 50 μm. (*P < 0.05, **P < 0.01, ***P < 0.001). **(D)** Representative multiplex immunofluorescence (mIF) images of HCC tissues confirming the spatial co-localization of MCM7 (nuclear) and CD8 (membranous). DAPI was used to stain the nuclei. Scale bars = 20 μm.

To validate our multi-omics findings, we utilized an in-house cohort of clinical HCC and adjacent normal tissues. The baseline clinicopathological characteristics of this validation cohort are summarized in **Supplementary Table 2**. Validation using these clinical tissues revealed a complex senescence-associated expression pattern. At the mRNA level, CDKN1A (p21) was significantly downregulated in tumors, while CDKN2A (p16) showed no significant difference (**Figure 7A**). At the protein level, Western blot analysis confirmed that both p21 and p16 were significantly downregulated in HCC tissues, concurrent with a marked upregulation of MCM7 (**Figure 7B**). Interestingly, while the overall tissue-level expression of p21 and p16 via IHC did not show significant differences, MCM7 exhibited strong nuclear accumulation and significantly higher expression in tumors (**Figure 7C**). To definitively evaluate the functional senescence status, we performed SA-β-gal staining and found SA-β-gal positive area was significantly reduced in HCC tissues (**Figure 7C**). Furthermore, IHC staining for lactyl lysine revealed a substantial elevation in HCC tissues (**Figure 7C**). We then performed mIF staining on clinical HCC tissues. Strikingly, mIF analysis confirmed the distinct co-localization of MCM7 and CD8, with MCM7 exhibiting strong nuclear expression within a subset of CD8-positive tumor-infiltrating lymphocytes (**Figure 7D**). Collectively, these results suggest that HCC tissues exhibit a profound suppression of functional senescence, coupled with strong proliferative licensing (MCM7) and metabolic rewiring (lactylation).

### MCM7 causally regulates cell proliferation and senescence markers in HCC cells

**Figure 8:**
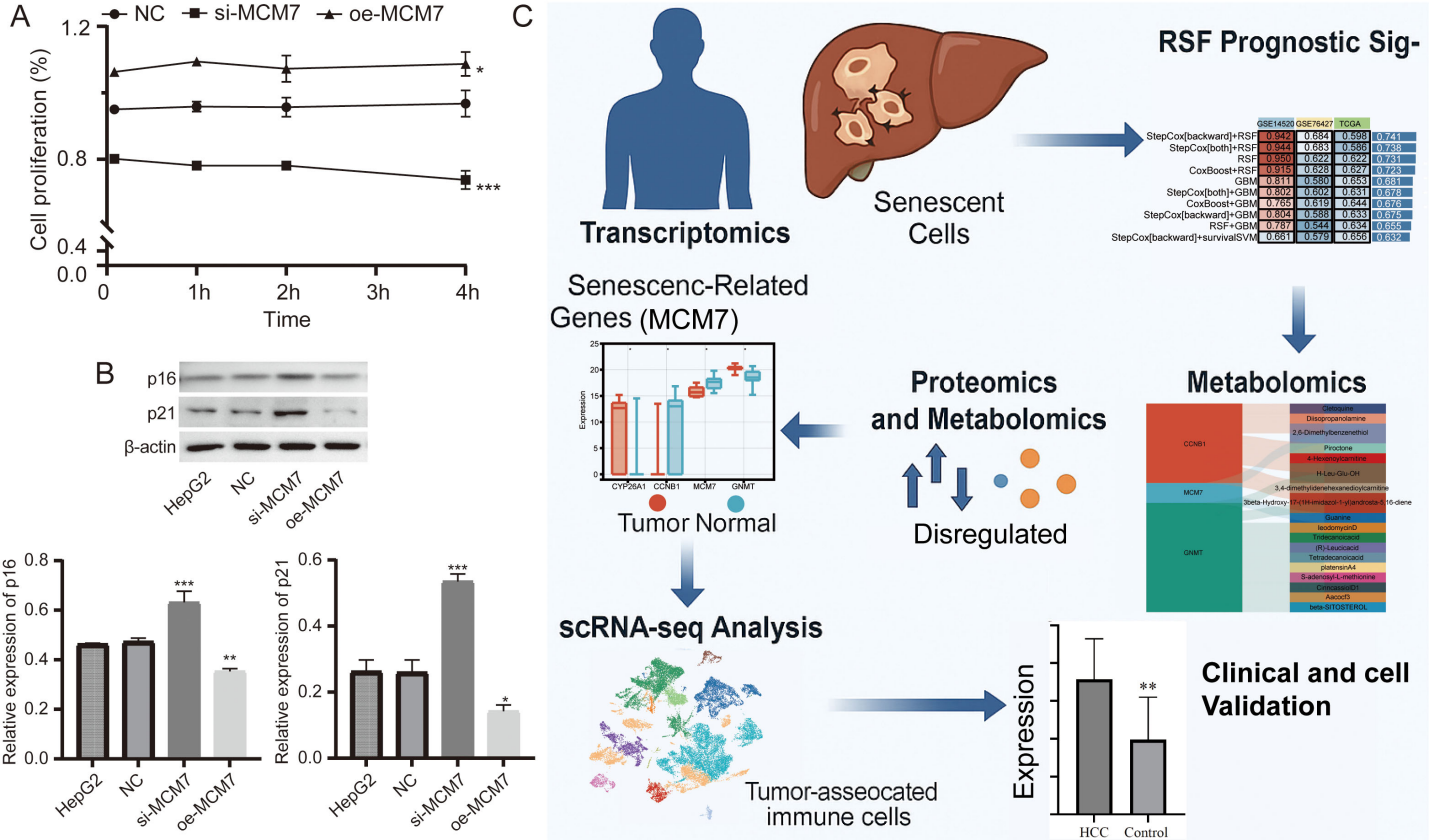
MCM7 functionally regulates cell proliferation and senescence markers *in vitro*. **(A)** CCK-8 assay showing the proliferation curve of HepG2 cells. (*P < 0.05, ***P < 0.001 vs. NC). **(B)** Western blot analysis and corresponding quantification of senescence markers p16 and p21 in HepG2 cells. (*P < 0.05, **P < 0.01, ***P < 0.001 vs. NC). **(C)** A schematic model illustrating the multi-omics landscape of senescence-associated progression in HCC.

We next performed *in vitro* functional assays by knocking down (si-MCM7) and overexpressing (oe-MCM7) MCM7 in the HepG2 HCC cell line. CCK-8 proliferation assays demonstrated that silencing MCM7 significantly inhibited the proliferative capacity of HepG2 cells, whereas MCM7 overexpression markedly accelerated cell proliferation (**Figure 8A**). Crucially, we evaluated the core senescence markers p16 (CDKN2A) and p21 (CDKN1A) via Western blotting. MCM7 knockdown dramatically upregulated the expression of both p16 and p21, MCM7 overexpression effectively suppressed p16 and p21 levels, facilitating senescence evasion and proliferative licensing (**Figure 8B**). Collectively, these functional studies provide direct causal evidence that MCM7 acts as a critical driver of HCC progression by suppressing senescence networks to promote tumor cell proliferation. Collectively, our multi-omics and clinical data established a robust correlation between MCM7 and the senescence-associated phenotype, and functional studies provide direct causal evidence that MCM7 acts as a critical driver of HCC progression by suppressing senescence networks to promote tumor cell proliferation (**Figure 8C**).

## Discussion

In this study, we integrated bulk transcriptomics, proteomics, metabolomics and single-cell RNA-seq to dissect how cellular senescence–related programs shape HCC. We built a 12-gene RSF prognostic model, and demonstrated that four of these genes (CYP26A1, CCNB1, MCM7, GNMT) are also altered at the protein level. Multi-omics integration further linked MCM7 to distinct metabolic states and specific immune-cell subsets. Together, these data support a model in senescence signaling activation without durable arrest, contributes to HCC progression through cell-cycle deregulation, metabolic rewiring and immune microenvironment remodeling.

Cellular senescence was originally defined as a durable cell-cycle arrest that limits the proliferation of damaged or oncogene-expressing cells and therefore acts as an important tumour-suppressive barrier (11). Within this framework, our observation that 58 cellular senescence–related genes are consistently dysregulated in HCC and enriched in cell-cycle, p53 signaling and amino-acid metabolism pathways is highly concordant with the dual role of senescence described in recent reviews (12). p21 and p16 were significantly downregulated in human HCC tissues, loss or suppression of p21 has been associated with increased proliferative capacity and poor outcome in multiple cancers (13). Such “senescence-associated” has been proposed as a mechanism by which cancer cells exploit senescence networks to enhance invasion, angiogenesis and epithelial-mesenchymal transition while avoiding permanent growth arrest (14).

Our 12-gene RSF model integrates classical cell-cycle regulators (e.g. CCNB1, MCM7) with metabolic enzymes (e.g. G6PD, GNMT), highlighting that senescence-related prognostic programs in HCC are not purely proliferative but also metabolic. The clinical translational potential of our RSF signature is further highlighted by its ability to predict immunotherapy response. Patients in the high-risk group exhibited significantly higher TIDE scores, indicating a greater potential for immune evasion and potential resistance to ICB.

High expression of MCM7 has previously been associated with shorter overall survival, intrahepatic metastasis and vascular invasion in HCC patients after resection, and multivariate analysis identified MCM7 as an independent prognostic factor (15, 16). Functional studies have shown that MCM7 enhances HCC cell proliferation and tumourigenicity *in vivo* by up-regulating cyclin D1 through MAPK signaling, thereby accelerating G1/S transition (17, 18). In the context of suppressed p21/p16, the simultaneous upregulation of MCM7 at transcript and protein levels may create a cellular landscape in which proliferative licensing overrides senescence enforcement, thus promoting tumor progression.

In contrast, GNMT is widely recognized as a tumour suppressor in liver carcinogenesis. GNMT-deficient mice spontaneously develop HCC and display aberrant Wnt activation and DNA methylation changes (19). The inclusion of GNMT as a protective factor in our model therefore agrees with its established tumour-suppressive role in HCC and underscores the importance of methyl-group and one-carbon metabolism in senescence-related tumour biology. In HCC, G6PD-driven pentose phosphate pathway activity supports NADPH production and redox homeostasis in many cancers, thereby buffering oxidative stress associated with oncogene-induced senescence (20). ESR1 and PPARGC1A, serve as core targets of Per- and polyfluoroalkyl substances in both HCC progression and prognosis (21). Thus, the 12-gene signature can be interpreted as a composite of cell-cycle accelerators and metabolic gatekeepers that together determine whether senescence programs remain tumour-suppressive or shift towards tumour promotion.

By overlaying proteomic data onto the transcriptomic signature, we confirmed that CYP26A1, CCNB1, MCM7 and GNMT are altered at both mRNA and protein levels in HCC. Our untargeted metabolomics analysis adds another dimension to this picture by revealing distinct metabolic states between tumour and normal tissues and by demonstrating strong correlations between key genes (CCNB1, MCM7, GNMT) and specific metabolites. Recent work has emphasized that metabolic reprogramming in senescent cells can reshape the tumour microenvironment and modulate immune responses to therapy (22). In particular, altered nucleotide and redox metabolism have been linked to the maintenance of senescent phenotypes (23). The tight association we observed between MCM7 and metabolites such as guanine is therefore consistent with a model in which replication-licensing machinery is functionally coupled to nucleotide metabolism and oxidative stress buffering in HCC.

A key novel aspect of our work is placing MCM7 within the immune landscape using scRNA-seq. We found that MCM7 is selectively enriched in tumour-derived CD8^+^ T cells and mast cells, rather than being restricted to proliferating hepatocytes. Reviews of the HCC immune microenvironment have highlighted that HCC is an inflammation-associated tumour with a profoundly immunosuppressive milieu that promotes immune tolerance and evasion (24, 25). More broadly, cellular senescence has been described as a double-edged sword in cancer immune surveillance: senescent cells can initially attract and activate immune cells to clear premalignant clones, but persistent SASP drives chronic inflammation and immunosuppression that facilitate tumour progression (26). Our study goes beyond mere correlation by providing direct functional evidence of MCM7’s role. In HepG2 cells, MCM7 knockdown significantly increased the expression of p16 and p21 while inhibiting proliferation, whereas its overexpression yielded the opposite effect. This suggests that MCM7 licenses DNA replication and promotes HCC progression by decoupling the cell cycle from senescence-enforcing signals (27, 28).

Our finding that MCM7 is up-regulated in CD8^+^ T cells within the tumour microenvironment raises the intriguing possibility that MCM7 marks a subset of proliferative or pre-senescent T cells at risk of functional exhaustion. By assigning cell cycle scores (S/G2M), we demonstrated that MCM7 expression in CD8+ T cells predominantly marks a highly proliferative subpopulation. We then performed mIF, which confirmed the spatial co-localization of MCM7 and CD8 at the protein level. This population likely represents a ‘proliferative exhausted’ T cell state, where intense clonal expansion coincides with an exhaustion trajectory under chronic antigen stimulation. Reviews of T-cell senescence in tumors emphasize that senescent T cells can retain cytotoxic potential but often produce high levels of inflammatory cytokines and display impaired clonal expansion, thereby contributing to dysfunctional immunity (29).

This study has several limitations. First and foremost, the sample sizes for our in-house proteomic and untargeted metabolomic analyses are extremely limited. Given the profound inter- and intra-tumoral heterogeneity characteristic of HCC, these small cohorts are underpowered to draw robust, generalizable conclusions regarding global protein and metabolic alterations. Consequently, these multi-omics components of our study should be strictly interpreted as preliminary, exploratory observations rather than definitive solid validations. While these exploratory protein and metabolic trends align well with our large-scale transcriptomic models (TCGA and GEO cohorts), large-scale, multi-center proteomic and metabolomic validation cohorts are urgently needed to substantiate these preliminary findings. Second, our work is largely correlative: while previous functional studies have established oncogenic roles for MCM7 in HCC, we did not experimentally manipulate these genes *in vivo* within this study. Third, the causal relationships among senescence activation, metabolic rewiring and immune dysfunction remain to be resolved; integrated CRISPR-based gene editing, lineage-tracing and immune-cell co-culture models will be needed to dissect these pathways. Finally, our multi-omics analysis is retrospective; prospective clinical trials will be required to evaluate whether senescence-targeted interventions, alone or combined with immunotherapy, improve outcomes in molecularly defined HCC subgroups.

## Conclusion

In summary, our multi-omics study demonstrates that cellular senescence–related programs in HCC are rewired in a way that couples cell-cycle drivers, metabolic circuits and immune microenvironment states. This senescence-escape framework unites cell-cycle deregulation, metabolic rewiring and immune microenvironmental changes, offering novel biomarkers (MCM7) and therapeutic avenues in HCC.

## Data Availability

The datasets presented in this study can be found in online repositories. The names of the repositories and accession numbers are as follows: GSE14520, GSE76427, GSE242889 (GEO); TCGA-LIHC (TCGA).

## Supplementary material

Supplementary Figure 1 Gene numbers included in the machine-learning prognostic models. Bar plot showing the number of genes incorporated into each of the model.

Supplementary Figure 2 Kaplan–Meier survival analysis of key prognostic genes in two independent cohorts. **(A)** Kaplan–Meier survival curves of G6PD, CCNB1, TACC3, MCM7, GNMT, ESR1, and PPARGC1A in the GSE14520 cohort. **(B)** Corresponding Kaplan–Meier analyses in the TCGA-LIHC cohort.

Supplementary Table 1 Differentially expressed proteins (DEPs) between HCC and paired normal tissues.

Supplementary Table 2 Clinical characteristics of 10 HCC validation cohorts.
