# Supplementary figures and images for: Machine learning-driven multi-omics integration uncovers a senescence associated molecular axis in HCC
Source: Front Immunol. 2026 May 8;17:1762222. doi: 10.3389/fimmu.2026.1762222 (PMC13195000; doi:10.3389/fimmu.2026.1762222)

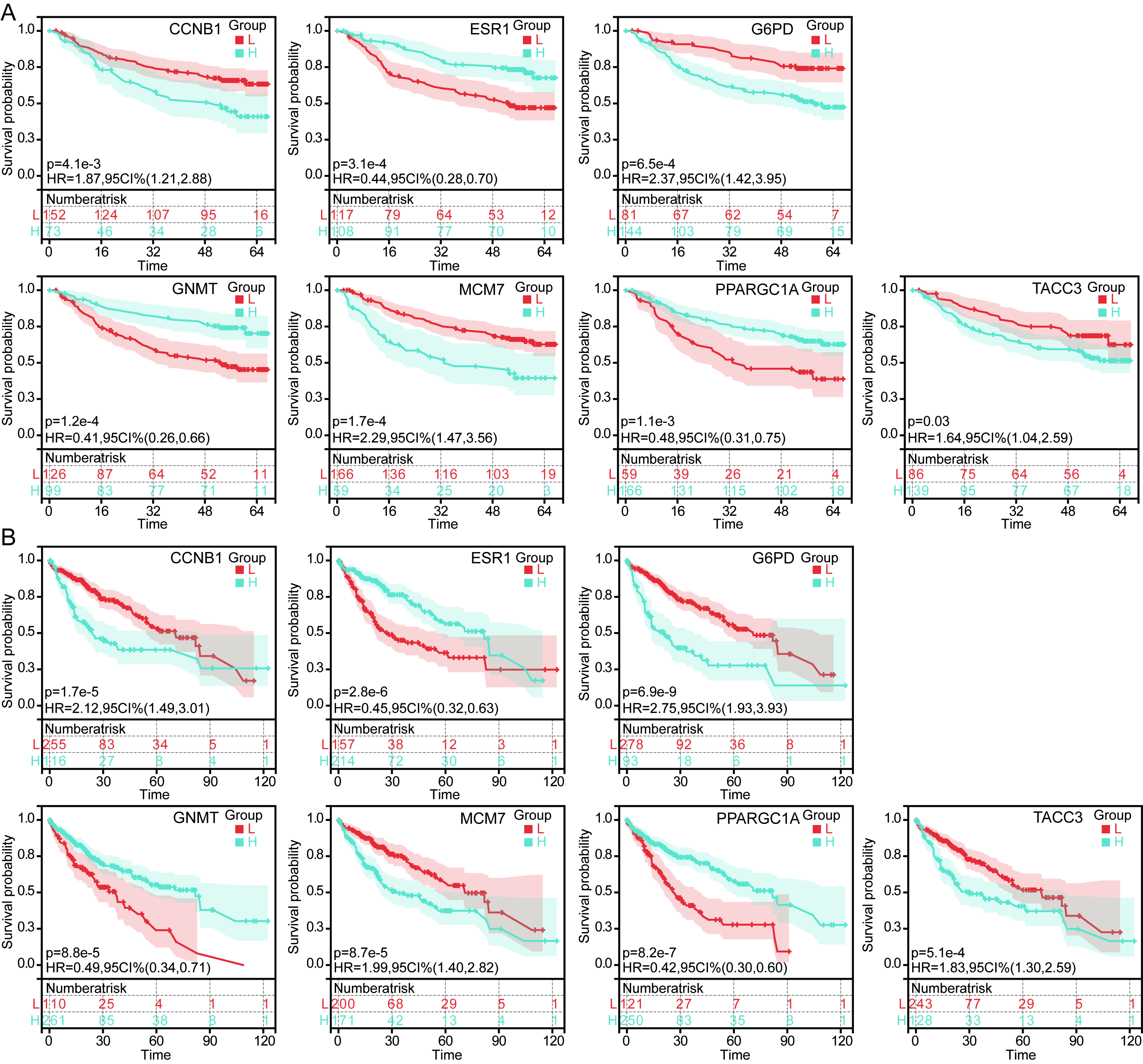

Supplement: Supplementary Figure 1 — Gene numbers included in the machine-learning prognostic models. Bar plot showing the number of genes incorporated into each of the model. [file Image1.tif]

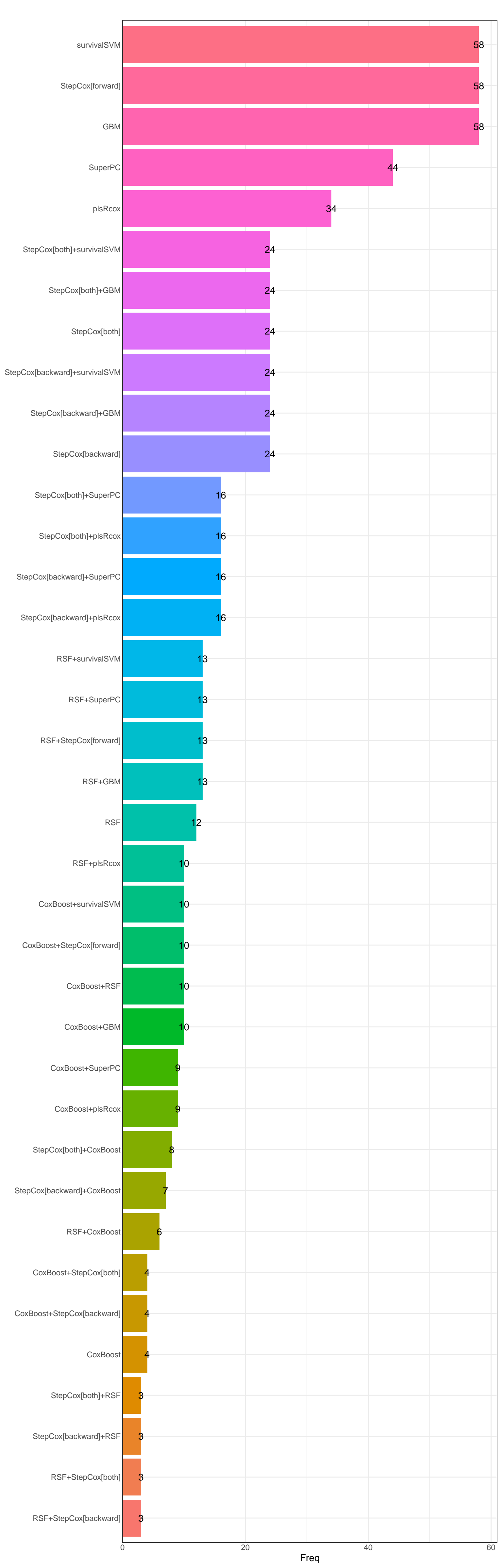

Supplement: Supplementary file 4 [file DataSheet1.pdf]
